# Biomimetic and Functional Nanomaterials for Molecular Imaging

**DOI:** 10.3390/pharmaceutics15061570

**Published:** 2023-05-23

**Authors:** Pedro Ramos-Cabrer, Jesús Ruiz-Cabello

**Affiliations:** 1 CIC biomaGUNE, Basque Research and Technology Alliance (BRTA), 20014 San Sebastian, Spain; 2 Ikerbasque, Basque Foundation for Science, 48009 Bilbao, Spain; 3 Biomedical Research Networking Center in Respiratory Diseases (CIBERES), 28029 Madrid, Spain; 4 Department of Chemistry in Pharmaceutical Sciences, Pharmacy School, University Complutense Madrid, 28040 Madrid, Spain

Welcome to this Special Issue of the journal *Pharmaceutics* entitled “Biomimetic and Functional Nanomaterials for Molecular Imaging,” which focuses on the exciting advancements in molecular imaging facilitated by biomaterials and nanotechnology. The development of non-invasive imaging technologies has revolutionized disease diagnosis, the study of the mechanisms of action, and the exploration of novel therapeutic approaches. In recent years, the convergence of biomaterials and nanotechnology has paved the way for innovative medical imaging techniques, propelling molecular imaging into a multidisciplinary discipline with tremendous potential.

Nanotechnology has played a pivotal role in transforming molecular imaging from a mere image acquisition process to a comprehensive field that combines sensitivity, selectivity, functional imaging, and the integration of therapeutic and diagnostic functions within molecular platforms (known as theranostics). By leveraging nanotechnology and existing imaging modalities such as magnetic resonance imaging (MRI), X-ray computerized tomography (CT), positron emission tomography (PET), and ultrasound imaging, the field of molecular imaging is becoming an essential technology in biomedicine.

Given the wide-ranging applications of biomaterials in biomedical research and, specifically, in the field of molecular imaging, this Special Issue aims to present noteworthy research and review articles that showcase the current state-of-the-art in biomaterial assessment, recent technological breakthroughs, and their functions and applications in molecular imaging.

We are thrilled to present a collection of insightful papers that exemplify the progress being made in the field. The articles selected for this Special Issue include:

1. “Synthetic Antiferromagnetic Gold Nanoparticles as Contrast Agents for MRI and CT” [1].
2. “Iron Oxide Conjugation for dual MRI and Fluorescent Imaging of Brain Tumors” [2].
3. “Divalent Manganese Ions for Cross-Linking and MRI Contrast in Intrathecal Injection of Hydrogel-Embedded Stem Cells” [3].
4. “Molecular Imaging for Myelin Quantification in Organotypic Cultures” [4].
5. “Iron Oxide-Bold Nanoparticles for In Vivo Imaging and Photothermal Therapy” [5].
6. “Quantification of Iron Oxide Nanoparticle Uptake in Mouse Brachiocelphalic Artery Atherosclerotic Plaque using T2-Weighted MRI” [6].
7. “Review of New Approaches in Nanomedicine for Ischemic Stroke” [7].

These papers highlight the diverse applications and significant contributions that biomaterials and nanotechnology bring to molecular imaging. From enhancing imaging contrast and targeting specific tissues to enabling therapeutic and cell interventions and quantifying disease progression, these studies exemplify the transformative potential of this interdisciplinary field.

We extend our gratitude to the authors for their valuable contributions and the reviewers for their diligent efforts in ensuring the high quality of the published work. Their collective efforts have made this Special Issue a comprehensive source of the latest advancements and insights in biomaterials and nanotechnology for molecular imaging.

We hope that this Special Issue serves as a valuable resource for researchers, clinicians, and stakeholders interested in the rapidly evolving field of molecular imaging. We anticipate that the findings presented here will inspire further exploration, collaboration, and innovative applications in the ever-expanding realm of biomaterials and nanotechnology for improved disease understanding, diagnosis, and treatment.

We hope that readers enjoy reading this Special Issue, and we hope that it will stimulate fruitful discussions and future breakthroughs in the fascinating field of molecular imaging.
